# The neutrophil-to-lymphocyte and monocyte-to-lymphocyte ratios are independently associated with clinical outcomes of viral encephalitis

**DOI:** 10.3389/fneur.2022.1051865

**Published:** 2023-01-11

**Authors:** Qiang He, Shuo Wang, Haoan Chen, Lili Long, Bo Xiao, Kai Hu

**Affiliations:** ^1^Department of Neurology, Xiangya Hospital, Central South University, Changsha, China; ^2^National Clinical Research Center for Geriatric Disorders, Xiangya Hospital, Central South University, Changsha, China; ^3^Clinical Research Center for Epileptic Disease of Hunan Province, Central South University, Changsha, China; ^4^Department of Neonatology, Xiangya Hospital, Central South University, Changsha, China; ^5^Faculty of Arts and Science, University of Toronto, Toronto, ON, Canada

**Keywords:** viral encephalitis, neutrophil-to-lymphocyte ratio, monocyte-to-lymphocyte ratio, prognosis, biomarker

## Abstract

**Background:**

The neutrophil-to-lymphocyte ratio (NLR) and monocyte-to-lymphocyte ratio (MLR) are used as prognostic biomarkers for many diseases. In this study, we aimed to explore the possibility of using ratios of NLR and MLR to predict the prognosis of viral encephalitis (VE).

**Methods:**

A total of 81 patients with an initial diagnosis of VE who were admitted to our hospital from January 2018 to January 2021 were retrospectively analyzed. A routine blood test within 24 h of admission was utilized to determine the ratios of NLR and MLR for each patient. The modified Rankin Scale (mRS) at 12 months after discharge was used to evaluate patients' clinical prognosis and the patients were divided into the group of good prognosis (mRS ≤ 1) and the group of poor prognosis (mRS ≥ 2) according to the mRS scores. Univariate and multivariable regression analyses were used to differentiate and assess independent prognostic factors for the prognosis of VE.

**Results:**

Neutrophil-to-lymphocyte ratio and MLR of the poor prognosis group were significantly higher than that of the good prognosis group. Multivariate logistic regression analysis results showed that NLR [odds ratio (OR): 1.421, 95% confidence interval (CI): 1.105–1.827; *P* < 0.05] and MLR (OR: 50.423, 95% CI: 2.708–939.001; *P* < 0.05) were independent risk factors for the poor prognosis of VE. NLR > 4.32 and MLR > 0.44 were suggested as the cutoff threshold for the prediction of the poor prognosis of VE.

**Conclusion:**

Neutrophil-to-lymphocyte ratio and MLR obtained from blood tests done at hospital admission have the potential to predict poor prognosis in patients with VE.

## Introduction

Viral encephalitis (VE) is one of the most common causes of sporadic encephalitis, which is caused by a neurotropic virus infection, such as herpes simplex virus, varicella–zoster virus, and enteroviruses (1). In the United States, the estimated incidence of VE has been reported as 7 per 100,000 person-years. In <50% of these cases, the specific virus infection could be identified (2). VE is characterized by various combinations of fever, headache, seizures, mental disorder, disturbance of consciousness, neurologic deficits, cerebrospinal fluid (CSF), and neuroimaging abnormalities (3). In recent years, although great progress has been made in antiviral therapies and supportive care, the prediction for prognosis of VE is still unsatisfactory, and certain extents of neurologic deficits or recurrent seizures are often followed after primary viral infection and encephalitis, which cause heavy economic and psychological burdens to patients (4, 5). Therefore, it is necessary to adjust the treatment plan through some readily available prognostic indicators for better outcomes.

Based on previous research on the pathophysiological mechanism of VE, it is recognized that the intracranial immune-inflammatory response activated by the virus has played a key role in the pathological damage of brain tissues (6, 7). A routine blood test is easily performed and can reflect the intracranial immune-inflammatory response to some extent (8). For example, the neutrophil-to-lymphocyte ratio (NLR) is defined as a simple ratio between the absolute count of neutrophils and the absolute count of lymphocytes, and it is an increasingly recognized systemic inflammatory response biomarker (9). In addition, NLR has also been proposed to function as an indicator of the general immune response to a variety of stress stimuli (10). Previous studies have shown that altered NLR has prognostic values in cryptococcal meningitis, tuberculous meningitis, and cerebrovascular disease (11–13). Moreover, it also has been demonstrated that abnormal NLR levels are associated with different prognoses in various tumors (14, 15). Recent studies have shown that the elevated level of NLR is a prognostic marker for several immune diseases of the neurologic system, including multiple sclerosis, neuromyelitis optica, and autoimmune encephalitis (16–18). The monocyte-to-lymphocyte ratio (MLR) is a novel clinically relevant biomarker of pathological inflammation similar to NLR. The MLR is calculated as the absolute count of monocytes divided by the absolute count of lymphocytes. In multiple sclerosis, MLR has recently been associated with disease progression and poor prognosis (17). However, no research has meaningfully explained the association between NLR and MLR and their prognostic values in VE yet. Therefore, we conducted a retrospective study of VE to differentiate and screen out whether NLR and MLR could be used as predictive biomarkers for VE prognosis.

## Methods

### Research subjects

Ethics approval for this study was obtained from the Ethics Committee of Xiangya Hospital of Central South University. We reviewed all medical records of patients diagnosed with VE who were admitted to the Department of Neurology, Xiangya Hospital of Central South University, between January 2018 and January 2021. Patients were eligible for inclusion only if they met all of the following inclusion criteria: (1) acute or subacute onset; (2) primary symptoms of fever, headache, seizures, mental disorder, disturbance of consciousness, and/or neurologic deficits; (3) head MRI indicating cerebral edema or focal or diffuse lesions; (4) lumbar puncture of CSF with normal or elevated pressure, normal or elevated white blood cell count, normal or slightly elevated protein levels, normal sugar and chloride levels, and no evidence of bacteria, tuberculosis, or fungal infection; and (5) if the antivirus treatment was effective. Patients were excluded if they met one of the following items: (1) suffered from other severe diseases, such as stroke, malignancy, and uremia; (2) diagnosed with encephalopathy secondary to metabolic, autoimmune, or sepsis conditions; or (3) were lost to follow-up.

### Data collection

We obtained the following clinical information: age, sex, white blood cell (WBC) count, neutrophils, monocytes, lymphocytes, NLR, MLR, erythrocyte sedimentation rate (ESR), C-reactive protein (CRP), cerebral spinal fluid (CSF), brain magnetic resonance imaging (MRI), and antivirus treatment delay (the time interval from onset to the initiation of antivirus treatment). Blood-related tests were recorded within 24 h of admission. NLR and MLR were defined as a simple ratio between the absolute count of neutrophils to the absolute count of lymphocytes (NLR), and the absolute count of monocytes to the absolute count of lymphocytes (MLR), respectively. Patients who received any kind of immunotherapy prior to admission were excluded. In our study, abnormal MRI result was defined as having findings that were described as cerebral edema or focal or diffuse lesions (19).

### Clinical outcomes

All patients had accomplished the follow-up of 12 months after discharge. The modified Rankin Scale (mRS) at 12 months after discharge was used to evaluate the clinical outcomes (16, 20). In our study, all patients were divided into two groups: the group of patients with an mRS score of 0–1 and a good prognosis; the group of patients with an mRS score of 2–6 and a poor prognosis.

### Statistical analysis

If the measurement data were normally distributed, it was presented in the form of mean ± SD and vice versa the median, IQR (interquartile range). Count data were expressed by frequency (in percentage). Chi-squared test (categorical variables), independent Student's *t*-test (normal distribution), and Mann–Whitney *U*-test (non-normal distribution) were used to analyze differences between the good and poor prognosis groups. We used univariate logistic regression analysis to determine correlations between selected covariates (including NLR and MLR) and clinical outcomes. Then clinical- or statistical-relevant variables from univariate analyses were used in the relevant multivariable logistic regression models to further analyze the possible association of NLR and MLR with clinical outcomes. We separately constructed three models to illustrate the stability of these relationships: Model 1 adjusted for none; Model 2 adjusted for sex and age; and Model 3 adjusted for sex, age, CSF results, brain MRI results, and antivirus treatment delay. The optimal cutoff values for VE prognostic indicators of NLR and MLR were determined by receiver operating curve (ROC) analysis. *P*-values of < 0.05 (two-tailed) were considered statistically significant. All statistical analyses were done using the SPSS IBM (version 25.0).

## Results

A total of 81 patients with VE met our inclusion and exclusion criteria, including 52 patients with a good prognosis and 29 patients with a poor prognosis. The median age of the 81 patients was 39 years, and 59.3% were men. CSF results and brain MRI results were abnormal in 40 and 28 patients, respectively. The median day of antivirus treatment delay was 5 days (Table 1).

**Table 1:** Basic characteristics of the poor prognosis group compared with the good prognosis group.

**Variables**	**Total**	**Good prognosis (*n* = 52)**	**Poor prognosis (*n* = 29)**	***P*-value**
Age (years) (median IQR)	39.0 (30)	33.5 (26)	56.0 (41)	**0.006**
Sex				0.701
Male	48 (59.3%)	30 (57.7%)	18 (62.1%)	
Female	33 (40.7%)	22 (42.3%)	11 (37.9%)	
WBC count, × 10^9^ cells/L (mean ± SD)	8.32 ± 2.87	7.68 ± 2.41	9.46 ± 3.29	**0.015**
Neutrophils, × 10^9^ cells/L (mean ± SD)	5.97 ± 2.83	5.15 ± 2.20	7.45 ± 3.26	**0.002**
Monocytes, × 10^9^ cells/L (median IQR)	0.60 (0.4)	0.60 (0.40)	0.60 (0.40)	0.885
Lymphocytes, × 10^9^ cells/L (median IQR)	1.50 (1.05)	1.70 (0.98)	1.20 (0.60)	**0.000**
NLR (median IQR)	3.80 (3.54)	2.77 (2.50)	6.23 (5.06)	**0.000**
MLR (median IQR)	0.38 (0.29)	0.35 (0.29)	0.50 (0.46)	**0.005**
ESR (median IQR)	29 (27)	23.0 (28.5)	35.0 (24.0)	0.588
CRP (median IQR)	4.40 (13.44)	7.50 (14.57)	4.05 (13.8)	0.952
CSF results				0.316
Normal	34 (45.9%)	20 (41.7%)	14 (53.8%)	
Abnormal	40 (54.1%)	28 (58.3%)	12 (46.2%)	
Brain MRI results				0.182
Normal	50 (64.1%)	36 (69.2%)	14 (53.8%)	
Abnormal	28 (35.9%)	16 (30.8%)	12 (46.2%)	
Antivirus treatment delay (days) (median IQR)	5 (6)	6 (6)	5 (8)	0.897

The bold values refer to *p*-values less than 0.05.

We present the basic characteristics of these patients according to their poor prognosis, as shown in Table 1. Patients in the poor prognosis group were older than those in the good prognosis group (*P* < 0.05), and there was no statistical difference in gender between the two groups (*P* > 0.05). There were significant differences between the good prognosis group and the poor prognosis group in age, WBC count, neutrophils, lymphocytes, NLR, and MLR (all *P* < 0.05). The NLR and MLR were significantly higher in the poor prognosis group than in the good prognosis group. In addition, no statistical difference was found in the monocytes, ESR, CRP, and CSF results, brain MRI results, and the antivirus treatment delay between the two groups (all *P* > 0.05).

In the results of the univariate logistic regression analysis, older age, higher WBC count, higher neutrophils, and lower lymphocytes were associated with poor prognosis (Table 2). Interestingly, antivirus treatment delay was indeed not significantly associated with patients' prognoses. Increasing NLR and MLR were related to greater odds of poor prognosis (OR 1.419, 95% CI: 1.168–1.722, *P* < 0.001; OR 17.662, 95% CI: 2.227–140.1, *P* = 0.007). To further investigate the association of NLR and MLR with prognosis, we used the multivariate logistic regression analysis method. By stepwise inclusion of covariates (sex, age, CSF results, brain MRI results, and antivirus treatment delay), we separately constructed three models to illustrate the stability of these associations. Our results showed that higher NLR (OR 1.421, 95% CI: 1.105–1.827, *P* = 0.006) and higher MLR (OR 50.423, 95% CI: 2.708–939.001, *P* = 0.009) had a significant linkage with poor prognosis, and these relationships were stable (Table 3), indicating NLR and MLR were independent risk factors for the poor prognosis of VE. The risk of poor prognosis increased with the increasing NLR and MLR. That is, for every one-unit increase in the NLR, there was a 42.1% increased risk for the poor prognosis of VE. For every one-unit increase in the MLR, there was a 49.4 increased risk for the poor prognosis of VE.

**Table 2:** Univariate analysis for clinical outcome in patients with VE.

**Variables**	**OR**	**95% CI**	***P-*value**
Age (years)	1.041	1.014–1.069	**0.003**
Sex (ref: male)	0.833	0.329–2.113	0.701
WBC count	1.253	1.053–1.491	**0.011**
Neutrophils	1.378	1.132–1.676	**0.001**
Monocytes	1.601	0.548–4.682	0.390
Lymphocytes	0.217	0.086–0,547	**0.001**
NLR	1.419	1.168–1.722	**<0.001**
MLR	17.662	2.227–140.1	**0.007**
ESR	1.004	0.986–1.022	0.679
CRP	0.996	0.982–1.010	0.564
CSF results	1.633	0.625–4.271	0.317
Brain MRI results	0.519	0.197–1.368	0.185
Antivirus treatment delay (days)	1.032	0.948–1.125	0.464

The bold values refer to *p*-values less than 0.05.

**Table 3:** Relationship between NLR and MLR and clinical outcome in patients with VE in different models.

	**OR (95% CI)**	

	**NLR**	**MLR**
Model 1	1.419 (1.168, 1.722)	17.662 (2.227, 140.100)
*P-*value	<0.001	0.007
Model 2	1.396 (1.135, 1.717)	21.49 (2.194, 210.445)
*P-*value	0.002	0.008
Model 3	1.421 (1.105, 1.827)	50.423 (2.708, 939.001)
*P-*value	0.006	0.009

**Figure 1:**
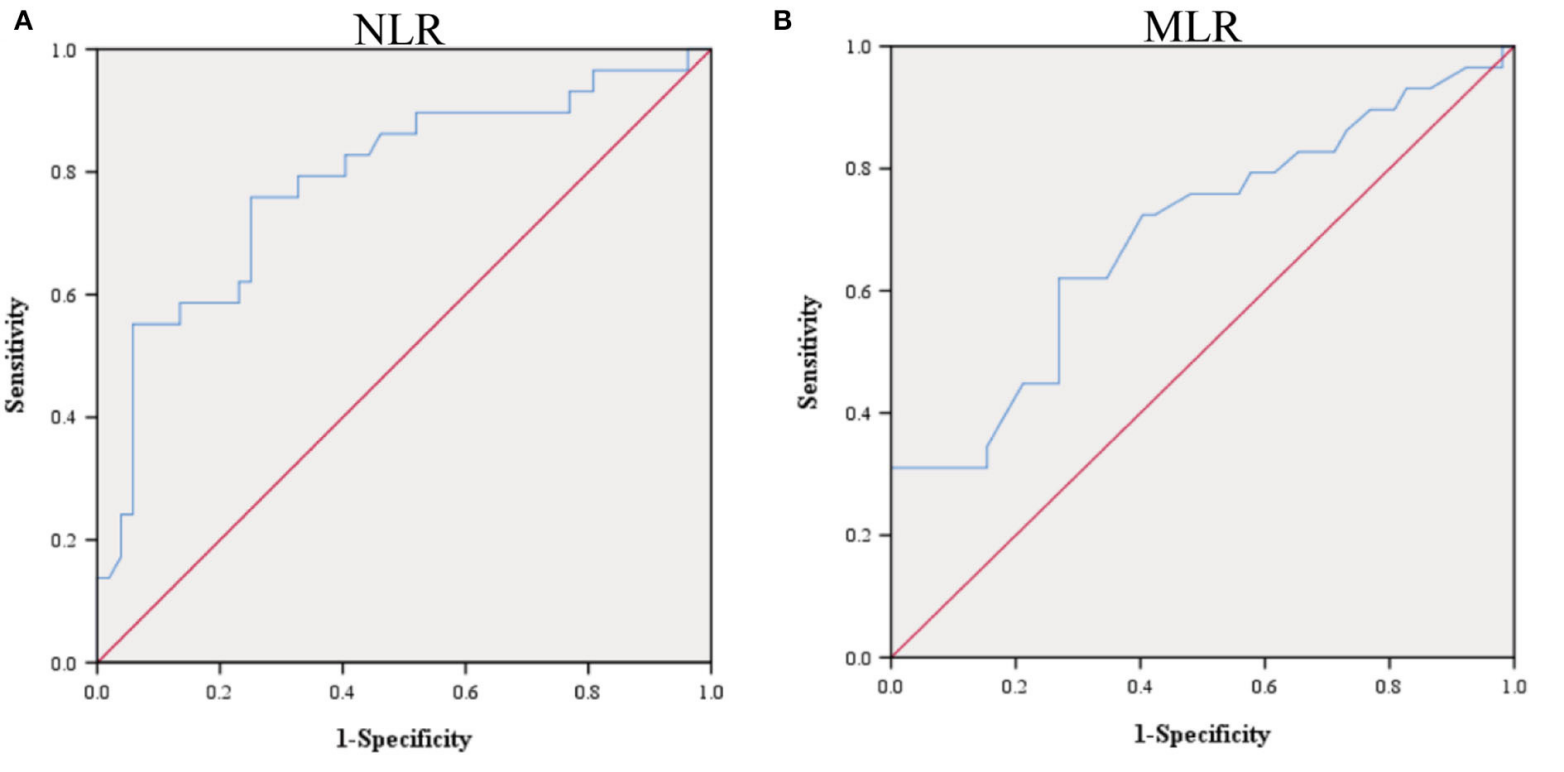
ROC curves of the predictive value of NLR **(A)** and MLR **(B)** for the poor prognosis of VE.

Given that NLR and MLR can be used to initially identify patients with a good or poor prognosis of VE, and that there were no uniform laboratory reference values, we calculated the optimal cutoff values by the ROC analysis. The ROC curves of NLR and MLR are shown in Figure 1. Areas under the curve (AUC) of NLR and MLR were 0.785 (95% CI: 0.676–0.895, *P* < 0.001) and 0.690 (95% CI: 0.565–0.815, *P* = 0.005), respectively, as shown in Figure 1 (Table 4). The optimal cutoff values were 4.32 (sensitivity, 0.759; specificity, 0.750) and 0.44 (sensitivity, 0.621; specificity, 0.731) for NLR and MLR, respectively.

**Table 4:** Receiver operating characteristic curve-related statistical indicators.

**Prediction**	**AUC**	**95% CI**	***P-*value**
NLR	0.785	0.676–0.895	< 0.001
MLR	0.690	0.565–0.815	0.005

## Discussion

In this study, we made a retrospective analysis of clinical data in patients with VE, mainly including laboratory tests, CSF tests, brain MRI examinations, and antiviral therapy. We focused on the role of peripheral blood immune cell counts and ratios as prognosis predictors for VE. The results of this study showed that NLR and MLR can be used as prognostic markers for VE.

As increasingly recognized biomarkers of the systemic inflammatory response, NLR and MLR have been widely used to predict the prognosis of many diseases. Some reports have demonstrated that abnormal NLR and MLR levels are related to the prognosis of various types of cancer. For example, Li et al. detected that both NLR and MLR are independent prognostic factors for tumor recurrence in patients with stage IIB cervical cancer (21). Song et al. found that NLR and MLR may become good biomarkers for predicting overall survival in patients with advanced gastric cancer (22). Chim et al. suggested high NLR and MLR before the treatment as biomarkers to identify patients with worse functional outcomes and overall survival of patients with glioma (23). In a retrospective study of myocarditis, NLR and MLR were associated with the severity of myocarditis and predicted length of hospital stay (24). Moreover, NLR and MLR are also related to the differential diagnosis and treatment of some diseases. Xu et al. revealed that NLR and MLR may be better predictive indicators for identifying prostate cancer (25). Those patients with higher NLRs had a higher chance of failing in the first-line treatment of autoimmune encephalitis (8). In patients diagnosed with tuberculous pleurisy, the peripheral blood MLR can be easily and effectively used to predict the treatment response (26). In addition, recent studies have also shown that increased NLR and MLR are related to higher rates of mortality in patients with COVID-19 (27, 28). Halmaciu et al. published an article in which they proved the prognostic impact of NLR > 6.97 and MLR > 0.54 in COVID-19 patients' death (29). Muresan et al. concluded that NLR > 9.4 and MLR > 0.78 are associated with mortality in a study conducted in a group of 889 patients with COVID-19 (30). From the above research results, NLR and MLR can well-reflect the occurrence and development of many diseases. In our study, NLR levels of >4.32 and MLR level of >0.44 were independent significant predictors of poor prognosis in patients with VE, which is broadly consistent with the findings of the aforementioned studies. In the present study and based on our findings, we have extended the previous scope regarding the prognostic role of NLR and MLR.

At present, it is recognized that the intracranial immune-inflammatory response activated by the virus has played an important role in the pathological damage of brain tissue in VE (6, 7). When the virus had invaded the central nervous system, the body first initiates innate immunity, and its main effector cells are neutrophils and monocytes. Neutrophils can defend against viruses by secreting many pro-inflammatory factors, such as tumor necrosis factor-alpha (TNF-α), reactive oxygen species (ROS), and interleukin 1 beta (IL-1β), and these pro-inflammatory factors also disrupt the integrity of the blood–brain barrier (31, 32). Monocytes are recruited to the sites of infection and partially differentiate into macrophages, which together function as neutrophils, as described earlier. Furthermore, macrophages also play a major role in initiating adaptive immunity, which is mainly mediated by T lymphocytes and B lymphocytes (33). B cells secrete specific antibodies that bind to free virus particles and block host cell infection (34). The primary role of T cells is to recognize and destroy virus-infected cells (35). Innate immunity can often delay the viral infection and create a time window for adaptive immune responses. It is the adaptive immune response that plays a key role in clearing the virus (36). Unlike individual neutrophil, lymphocyte, and monocyte counts, NLR and MLR have less variability in healthy populations and can more accurately reflect the body's immune-inflammatory status under pathological conditions. As a result, we speculate that higher NLR and MLR can imply an imbalance of innate and adaptive immune homeostasis, which would further amplify the immune-inflammatory cascade. It also helps to explain why higher NLR and MLR are associated with poor prognosis in VE.

In line with expectations, older age was shown and related to poorer functional outcomes, which may be the reason for the corresponding decrease in immunity with increasing age. Furthermore, we found that antivirus treatment delay, a well-recognized marker of poor prognosis, was not associated with poor prognosis in VE. This may reflect updates in VE diagnosis and treatment strategies over time compared to previous studies. It is common that when suspected VE is diagnosed, empiric antiviral therapy is initiated immediately, thus, the delay in antiviral therapy is getting shorter and shorter. In addition, we speculated that there was no significant difference in initial disease severity between the good and poor prognosis groups, so a shorter delay in antiviral treatment was not a factor in determining patient outcomes.

Our study mainly aims to explore the prognostic value of NLR and MLR in patients with VE. NLR and MLR are commonly tested and have a low cost when compared to other inflammatory markers, including D-dimers, interleukin-6, and fibrinogen. Their use in medical practice allows for better stratification of risk groups and the establishment of appropriate therapeutic management, thus improving the progression of patients with VE. However, there are several limitations to our study. First, because this study was retrospective, it is difficult to control for confounding factors. Second, there is an inevitable risk of bias as the present study was only conducted in a single center with a small sample size. Third, the positive rate of clinical detection and isolation of viruses is very low. After comprehensive consideration, we did not include virus detection or isolation in the diagnostic criteria. Last, since other types of infections and autoimmune diseases can affect the NLR and MLR, we excluded patients with these comorbidities. Hence, the result of this study may not be appropriately applied to all patients with VE.

In summary, our data indicate that NLR and MLR on initial hospital admission blood tests may have predictive value for poor prognosis in patients with VE. This finding can help clinicians to early identify those critically ill patients with poor prognoses to optimize clinical treatment decisions, thereby improving patients' outcomes to a certain extent.

## Data availability statement

The original contributions presented in the study are included in the article/supplementary material, further inquiries can be directed to the corresponding author.

## Ethics statement

The studies involving human participants were reviewed and approved by the Ethics Committee of Xiangya Hospital of Central South University. The patients/participants provided their written informed consent to participate in this study.

## Author contributions

KH designed the study, reviewed, and revised the manuscript. QH collected data and drafted the manuscript. SW, HC, LL, and BX reviewed and revised the manuscript. All authors read and approved the final manuscript.
